# Why the war on drugs in sport will never be won

**DOI:** 10.1186/s12954-015-0087-5

**Published:** 2015-11-10

**Authors:** Aaron C. T. Smith, Bob Stewart

**Affiliations:** College of Business and Law, RMIT University, Melbourne, Australia; College of Sport and Exercise Science, Victoria University, Melbourne, Australia

**Keywords:** Doping, Sport, Drugs-in-sport, Performance-enhancing drugs, Harm reduction

## Abstract

Recent exposes of drug use in sports suggest that doping might be more problematic than doping-control test results reveal. A zero-tolerance (ZT) model, which aims to eliminate the use, has dominated the thinking of sport’s policy makers over the last 15 years. In light of the limitations associated with ZT-based policy, we propose an alternative policy, one based on controlled use and harm reduction principles. We argue that substance control policies underpinned by harm reduction (HR) principles of social utility and public value will deliver superior social outcomes. First, a harm reduction approach better accommodates the competitive realities of sports and the impact of elite sports’ emphasis on performance at all costs. Second, HR prioritises athlete welfare over sport and brand reputation. Finally, while appreciating the regulatory and risk management responsibilities of sports’ governing bodies, the HR model offers greater space to the athlete’s right to privacy, and right to personal autonomy.

## Background

We begin our commentary of the drugs in sport problem by asserting that drug use is both endemic in modern society and a feature of contemporary sport. We also suggest that drug use in sport has few ‘black and white’ features, as its critics tend to suggest. Rather, the contextual complexities associated with drug use in sport make its management problematic. As a result, the rationale for, and mechanisms of, drug control remains a subject of heated debate. The prevailing policy is orchestrated by powerful global-sport authorities like the International Olympic Committee (IOC), the World Anti-Doping Agency (WADA), and international sport federations, which claim that drug use is cheating and should be eliminated through the imposition of severe punishments. However, we argue that the current policy has neither been successful in eliminating doping in sport, nor effective in protecting the health of athletes.

The scope and scale of doping in ‘tested’ sport remains unclear. Numerous studies suggest that prevalence rates could be much higher than doping control tests reveal [1, 2]. One study based on a combination of questionnaires and statistical models of plausible biological anomalies estimated a figure of 14–39 % compared to the 0.5–2 % level of positive doping control tests [3]. Athlete and coach surveys suggest higher rates of usage as well, although respondents tend to identify doping in their peers than admit their own personal use [4, 5]. When asked about personal use—either through questionnaires or interviews—the respondent results are closer to control test levels, with higher levels of illicit drug use than of performance-enhancing substance use [6]. Doping prevalence may be even higher in serious recreational and fitness sports [7], while usage by adolescents appears to be growing [8]. The use of medications by elite athletes has also been shown to reach higher levels than the non-sporting public [9].

One proposed solution to the doping problem involves even more rigorous testing protocols. They include greater frequency of random doping analyses, enforced medical follow-ups, stronger legislation against the possession of doping substances, and harsher penalties for athletes who use the substances [10]. In contrast, we propose an alternative approach by focusing on the protection of athlete health, the retention of their civil rights, and the reduction of drugs’ negative social impacts. This harm reduction model presents an essentially utilitarian position, where ethical judgement and moral certitude are replaced by the practicalities of managing the multiple potential harms associated with elite sport. Our position maintains that the current WADA policy of zero tolerance will neither extinguish doping in sport nor protect the health and well-being of athletes.

The evidence provided by athletes themselves supports our claim. In our studies [11, 12] and others [13], many elite athletes make it clear that they would try any performance-enhancing substance as long as it is not banned. Safeguarding health plays a negligible role in the decision-making process. Athletes do not rely on a set of immutable moral boundaries. In fact, ‘clean’ athletes often use performance-enhancing substances that do not appear on the official World Anti-Doping Agency (WADA) prohibited list [14].

But, does it matter that athletes underplay the significance of health or morality as long as they remain ‘list’ compliant? Yes, it does matter, because punitively driven deterrence does not work, especially when the motivation for substance use comes from the pursuit of superior performance. Consider the teenage Olympic-level gymnast in one of our studies who consumed analgesics by the handful to control her chronically agonising joint pain [11]. An ever-vigilant scanner of the banned-substance list, she reported her delight as a stronger painkiller became available when it was removed from prohibition. Our gymnast, like the cyclists in another of our studies [15], also consumed significant quantities of caffeine but still viewed herself as ‘clean’. Then, there was the case of a wheelchair powerlifter sanctioned by his governing body under a therapeutic exemption to use nandrolone decanoate to rehabilitate a torn pectoralis major. Our results show that athletes experience transitional pressure to use more substances, even when remaining ‘clean’. To speculate in the absence of evidence, it is also possible that some athletes employ higher dosages of normally banned substances while permitted to do so under the umbrella a therapeutic exemption.

Although so-called gateway theories may deserve the critical scrutiny they have recently received in relation to recreational and illicit drugs [16, 17], some early work in sport suggests that doping or substance ‘creep’ should be taken seriously, especially when considered in light of emerging evidence connecting favourable perceptions of performance-enhancing substance benefits with their use in elite sport [18, 19]. For example, supplement users hold more permissive attitudes to banned doping in sports than those not using supplements, where supplement users are three and a half times more likely to practice banned doping than athletes not using supplements [20]. Barkoukis et al. concluded, for example, that the use of nutritional supplements is associated with biased reasoning in favour of doping [21]. In fact, young elite athletes who declare that supplementation is essential for sporting success are more likely to condone doping [22].

Another important study indicated that body dissatisfaction, weight change behaviours, and supplement use are related to more lenient attitudes towards sport doping in adolescents [23]. A similar study reported a relationship between the use of protein, creatine, and anabolic steroids, where the use of each former substance provided a statistical predictor of the next step in the hierarchy of drug use [24]. Elite athletes report that in order to reach the highest levels of performance, it is necessary to go beyond ‘naturally evolved talent’ through a combination of advanced training, coaching, supplements, and substances [25]. Even athletes from club-level sport who have rejected the use of banned substances seem to recognise that in order to effectively transition to the next level, some additional substance use may be required [26]. Similarly, our own research shows that while mid-level performing athletes nearly always fall short of using banned substances, they understand that in order to achieve national or international success, additional substance use is essential [11]. Furthermore, athletes’ attitudes to banned substances are in part shaped by the attitudes and practices of fellow sport participants. Favourable views about substance efficacy and appropriateness are likely to undermine effective regulation by normalising their use [18, 19]. These studies have highlighted the ways in which peer-pressure in the form of ‘social networks’, external ‘facilitators’, and ‘inhibitors’, influence supplement and drug use throughout an athlete’s sporting career [18].

In a mind set that so easily accommodates shifts in what constitutes a banned substance, many athletes experience substance ‘creep’ over their competitive careers. Pain, sacrifice, and psychological trauma are normal constituents in the elite athlete’s routine; risk and health problems are part of the game. The threat of sanction, however severe, pales against a cost-benefit algorithm where failure is just as unpalatable as victory is compelling [27]. And that is before any economic incentives add impetus. To compound matters, elite athletes use prohibited performance-enhancing substances to bolster training and recovery as much as to supplement in- competition performance, leaving only out-of-season testing to sidestep. Studies have also revealed that it is possible for athletes to successfully use micro-dosing strategies in order to pass tests [28].

Vigilant testing and heavy sanctions stimulate athletes to use more dangerous substances and combinations for both masking and performance purposes. Our most recent research, as well as other meta-studies, shows that elite sport presents a special problem because its performance demands encourage, and perhaps even impel, the experimental use of substances [13, 14, 29]. For example, in one study, banned athletes reported that a motivation for doping was to keep pace with competitors and ensure the financial rewards of success [30], a form of rationale Kirkwood labelled ‘defensive doping’ [31]. Perhaps more worrisome is Fincoeur et al.’s caution that the pursuit of substance suppliers by the drug regulators can potentially drive athletes to secure illegitimate and uncontrolled sources, and thus exacerbate the banned substance-use problem [32].

## From compliance to health

In 2004, WADA introduced its global anti-doping code. With the International Olympic Committee’s endorsement, it soon became the policy benchmark. Three key objectives underpin WADA’s mission and policy initiatives: first, to protect the athletes’ fundamental rights to participate in drug-free sport; second, to promote health, fairness, and equality for athletes worldwide; and finally, to ensure harmonised and effective anti-doping programmes at the international and national levels incorporating standardised approaches to detection, deterrence, and prevention. The code contains a list of banned substances including performance-enhancing drugs like EPO, human growth hormone, anabolic androgenic steroids, the more powerful anti-inflammatory drugs and stimulants, and a range of non-performance enhancing, illicit drugs like cannabis, ecstasy, and cocaine. Exemptions exist in the code for athletes who can demonstrate a legitimate therapeutic purpose for a banned substance. In these instances, athletes with documented medical conditions like asthma can request a therapeutic use exemption from their national anti-doping agency and national sport governing body.

Under WADA’s policy, drugs that enhance performance are immediately corralled, since using drugs to help secure a winning edge constitutes cheating, which as noted before, will not be tolerated. Second, drugs that constitute a risk to the athlete’s health also fall into the anti-doping net. According to WADA, sport organisations hold a duty of care to the athletes who participate in their competitions and, as a result, must be protected through prohibitions on substances incurring health risks. The third and final element of the WADA anti-doping code is more contentious since it outlaws any drug that violates the ‘spirit-of-sport’.

According to the code, the spirit-of-sport encapsulates the ideals of Olympism, the celebration of the human spirit, fun, and joy, courage, teamwork, excellence in performance, respect for the rules and other participants, dedication and commitment, character and education, community and solidarity, ethics, fair play, and honesty. These values have been conflated into the initial WADA slogan of ‘play-true’ and its current mantra of ‘drug-free’ sport. If a drug meets two of the above three criteria, it will be listed as a banned substance.

Under the WADA code, drugs like EPO, human growth hormone, steroids, and stimulants both enhance performance and constitute a health risk, and therefore remain banned. WADA policy also places illicit drugs under the banned-substance umbrella. However, unlike the all-year-round ban on performance-enhancing drugs, the illicit drug ban only applies to in-competition or in-season use. While cannabis, ecstasy, and cocaine do not enhance performance, they do introduce health risks. Crucially, because their illegal use undermines the spirit-of-sport, they too are banned. In fact, any illicit drug is, according to WADA, contrary to the spirit-of-sport since it diminishes the good name and public image sport commands. Caffeine, however, no longer appears on the banned list. Although caffeine improves performance, it is not illegal, does not incur health concerns, and fits the play-true requirement. Neither is alcohol nor tobacco/nicotine a major problem under the WADA Code since they also fit the play-true requirement in that they do not for the most part improve sporting performance, remain freely available to adults, and form an integral part of sporting club culture. Yet, studies reveal that athletes binge-drink and use recreational drugs to alleviate the pressure accumulated from demanding seasons of abstinence and stress [33]. The use of analgesics and painkillers also remains unclear especially when under legitimate prescription by medical practitioners.

Athletes occupy a world where drug use is embedded in community culture and practice. While large numbers of drugs are misused and produce significant social costs, they also provide the community with a better quality of life. A cursory look at mainstream drug use statistics shows that drug use is not an aberrant behaviour confined to a problematic subculture of deviants and misfits [34]. While the social burden of illicit drug use is undeniably severe, conflating the so-called war on drugs with a war on doping may risk ignoring the unique elite sporting context. Importing illicit drug policy into the sporting arena assumes that performance doping mirrors recreational and addictive drug behaviours, which doping undermines sport and morality in a similar way as criminal drug trafficking, and that doping decisions can be influenced by rational evaluations of the risk of severe penalties [35, 36]. It might also be ambitious to expect elite athletes to eliminate their use of drugs when society as a whole relies on drugs to help its members cope with the pressures and tensions of daily living and to help them feel psychologically and physically better.

Such mixed messages become compounded when we assume that using an over-the-counter drug with significant side effects is acceptable, but the use of an illicit drug with no greater side effect is not only taboo but also indicative of a moral failing. The message can be further confused when officials, journalists, and fans not only demand that athletes always perform at their best but also remind them that failure will be publically scrutinised. In analysing a series of case studies, Carstairs exposed the complex and often contradictory responses to doping expressed through the popular media, message boards, and polls [37]. Athletes who have failed drug tests can receive sympathy and condemnation simultaneously.

## An alternative policy

We contend that the primary principle of sound drug management in sport should be HR. In the context of sport, the HR approach illuminates three principles. First, drug use is not just a sporting matter nor is it a criminal or legal matter. Instead, drug use in sport constitutes a serious social issue [38]. Second, HR obviates the need for any form of moral certitude [39]. Instead, it accepts that drug use exists in sport and will never be completely eliminated. Third, although HR does not condone the use of drugs in sport, it acknowledges that when it does occur, policy makers have an obligation to develop public health measures that reduce drug-related harm to all athletes, irrespective of their status or ambition [40]. For example, policies that exclusively pursue the elimination of doping do not account for high or low risk use. Conversely, some evidence indicates that harm reduction polices providing education, private support, and rehabilitation, lower the social costs and cultural damage associated with substance use [41]. The key issue for HR therefore has less to do with the short-term brand equity and credibility that might be tarnished by a drug use or drug trafficking incident, and more to do with the long-term best interests of sport participants.

Unlike current drug control policies, HR is not about obstructive policing, incessant testing, onerous investigation, and severe sanctioning. Instead, it focuses on building structures and systems that deliver a number of harm reduction outcomes including for example: (1) the creation of a playing environment where safety and effective harm management are strategic priorities; (2) a drug supply and distribution system that is regulated through the direct involvement of physicians and pharmacists; (3) the design of promotional campaigns that educate athletes about the risks associated with various substances; (4) the early intervention of medical support where damage to oneself or to others has occurred through some form of drug use; (5) the availability of broad-based drug rehabilitation and counselling services that allow athletes to remediate their high risk behaviours; and (6) a transparent listing or register of the drugs used by all sporting bodies and athletes. In this context, regulation becomes useful only in so far as it lessens the potential harm of participants.

## Conclusions

Despite the pressures on serious athletes to use substances as they move along the performance pathway, our data show that mid-tier athletes practice considerable self-determination in selecting which substances to utilise [14]. The same thinking that leads individuals to improve their equipment technologies, strengthen their training programmes, and take nutritional supplements, also leads them to evaluate the benefits and costs of not only pushing the boundaries by using complex, high-dose multi-mix supplements but also by crossing the boundary into the realm of banned drug use. Yet we know little about these cogitations, when athletes are most vulnerable during their life cycles and the decision-making that emerges as a consequence. These findings suggest that sports officials have a window of opportunity for guiding serious athletes into safe and legal substance use through well-timed educational campaigns that deliver non-judgmental analyses of the strengths and weaknesses of substance use in all of its intricacies. We should also not forget that the majority of published studies have focused on clinical populations and case studies which tend not to address the supra-therapeutic regimens and complex pharmacology employed by athletes. Moreover, the most dangerous and prolific usage of substances can be found in groups primarily interested in recreational performance and image-enhancement, be it to build muscle, strip fat, or iron-out cellulite [42, 43].

Although HR is especially controversial and ‘messy’ [44] in a sporting context since it appears to condone practices that may be illegal and apparently unfair, it accepts the fact that drugs will always be part of a risky and tilted playing field full of moral ambiguity. Equally, it also allows for a stronger platform of education and social marketing and the provision of personnel and facilities that ensure a safe and protective sport environment where athlete welfare holds sovereign.

A zero-tolerance approach to drug use in sport leverages a strong sense of moral certitude, but it has not worked [45, 46]. In an interview with BBC in July this year, WADA Director General David Howman claimed that more than 10 % of elite athletes were doping. The greatest area of concern, he noted, was the level of up and coming athletes trying to get what he called a ‘breakthrough’, which made them more susceptible to substance abuse. Harm reduction approaches will never eliminate use, but they deliver a humane service to a cohort of talented performers who deserve a safe and supportive workplace in which to ply their highly skilled and heavily sought-after trade.

We have one policy model driven by a fundamentalist concern for punishment, zero tolerance and abstinence, and another underpinned by an idealistic concern for athlete autonomy, agency, and safety. Each of these positions has its strengths and weaknesses, but we need to determine which model ensures sports’ integrity the most, which one delivers the best outcomes for players and athletes, and which one offers the opportunity for sports’ other stakeholders to also benefit. However, these policy options are difficult to precisely evaluate, since subjectivity and bias inevitably get in the way of an impartial analysis, even where a lot of objective evidence has been compiled. Zero tolerance is likely to deliver lower levels of use, but it will impact on a player’s civil liberties, and the overall harms to the athletes may not necessarily be lowered since banned substance use presents only one of many catalysts for harm to occur in and through sport. A harm reduction approach will deliver greater autonomy to athletes, while pro-actively seeking to contain the damage to users and the people around them.
